# Automated Quality Control Solution for Radiographic Imaging of Lung Diseases

**DOI:** 10.3390/jcm13164967

**Published:** 2024-08-22

**Authors:** Christoph Kleefeld, Jorge Patricio Castillo Lopez, Paulo R. Costa, Isabelle Fitton, Ahmed Mohamed, Csilla Pesznyak, Ricardo Ruggeri, Ioannis Tsalafoutas, Ioannis Tsougos, Jeannie Hsiu Ding Wong, Urban Zdesar, Olivera Ciraj-Bjelac, Virginia Tsapaki

**Affiliations:** 1 Department of Medical Physics and Clinical Engineering, University Hospital Galway and Physics, School of Natural Sciences, University of Galway, H91 TK33 Galway, Ireland; christoph.kleefeld@universityofgalway.ie; 2 National Cancer Institute, Mexico City 07760, Mexico; jorge.castillo.mex@gmail.com; 3 Instituto de Física, Universidade de Sao Paulo (USP), R. do Matao, 1371-Butanta, São Paulo 05508-090, Brazil; pcosta@if.usp.br; 4 European Georges Pompidou Hospital, 75015 Paris, France; isabelle.fitton@aphp.fr; 5 National Cancer Institute, University of Gezira, Wad Madani 11111, Sudan; a.y.a.m.nci@gmail.com; 6 National Institute of Oncology, 1122 Budapest, Hungary; pesznyak.csilla@oncol.hu; 7 Fundación Médica de Río Negro y Neuquén-Leben Salud, Cipolleti R8324, Argentina; rruggeri@lebensalud.com; 8 Hamad Medical Corporation, Doha 3050, Qatar; itsalafoutas@hamad.qa; 9 University Hospital of Larissa, University of Thessaly, 41110 Larissa, Greece; tsougos@med.uth.gr; 10 Department of Biomedical Imaging, Faculty of Medicine, University of Malaya, Kuala Lumpur 50603, Malaysia; jeannie_wong80@um.edu.my; 11 Institute of Occupational Safety, 1000 Ljubljana, Slovenia; urban.zdesar@zvd.si; 12 Division of Human Health, International Atomic Energy Agency, 1220 Vienna, Austria; o.ciraj-bjelac@iaea.org

**Keywords:** chest radiography, image quality, remote, automated, quality control, quality assurance

## Abstract

**Background/Objectives**: Radiography is an essential and low-cost diagnostic method in pulmonary medicine that is used for the early detection and monitoring of lung diseases. An adequate and consistent image quality (IQ) is crucial to ensure accurate diagnosis and effective patient management. This pilot study evaluates the feasibility and effectiveness of the International Atomic Energy Agency (IAEA)’s remote and automated quality control (QC) methodology, which has been tested in multiple imaging centers. **Methods**: The data, collected between April and December 2022, included 47 longitudinal data sets from 22 digital radiographic units. Participants submitted metadata on the radiography setup, exposure parameters, and imaging modes. The database comprised 968 exposures, each representing multiple image quality parameters and metadata of image acquisition parameters. Python scripts were developed to collate, analyze, and visualize image quality data. **Results**: The pilot survey identified several critical issues affecting the future implementation of the IAEA method, as follows: (1) difficulty in accessing raw images due to manufacturer restrictions, (2) variability in IQ parameters even among identical X-ray systems and image acquisitions, (3) inconsistencies in phantom construction affecting IQ values, (4) vendor-dependent DICOM tag reporting, and (5) large variability in SNR values compared to other IQ metrics, making SNR less reliable for image quality assessment. **Conclusions**: Cross-comparisons among radiography systems must be taken with cautious because of the dependence on phantom construction and acquisition mode variations. Awareness of these factors will generate reliable and standardized quality control programs, which are crucial for accurate and fair evaluations, especially in high-frequency chest imaging.

## 1. Introduction

Radiography is an essential and low-cost tool in healthcare, providing diagnostic information quickly and efficiently, aiding in the timely management and treatment of various medical conditions [1,2]. Plain chest radiography is the most commonly performed X-ray examination [2,3]. It provides images of the thorax and surrounding structures, which are useful for identifying abnormalities in lungs. It is therefore used in pulmonary medicine for the initial assessment and management of patients with various pulmonary conditions, as well as for supporting clinical decision making [4,5]. The importance of chest radiography is underscored by the broad spectrum of indications. The main indications for chest radiography include, but are not limited to, the following [1]: (i) evaluation of signs and symptoms potentially related to the respiratory, cardiovascular, upper gastrointestinal, and thoracic musculoskeletal systems; (ii) staging of neoplasms as a complementary method to computed tomography imaging; (iii) follow-up on known thoracic disease processes when clinically indicated; (iv) monitoring of patients with life-support devices or of patients who had undergone surgery; (v) pre-operative assessment; and (vi) screening for diseases as required by public health regulations.

Chest radiography is one of the few examinations for screening employing ionizing radiation. It allows for the early detection of lung diseases, often before symptoms become apparent [6]. Radiographs can reveal small nodules or masses, which can then be investigated further with additional imaging or biopsies to determine their nature. Another important contribution of radiography lies with the monitoring of disease progression and the monitoring of treatment response, particularly in patients with chronic lung diseases such as chronic obstructive pulmonary disease or interstitial lung disease. Radiography is also essential for evaluating the effectiveness of treatments in patients undergoing chemotherapy or radiotherapy for lung cancer where sequential radiographs can show reductions in tumor size and, thus, indicating a positive response to treatment. Finally, in acute settings such as emergency departments, chest radiographs are indispensable for the rapid diagnosis of life-threatening conditions, such as pneumothorax, pleural effusion, and acute infections like pneumonia [7,8].

Imaging of thoracic structures is technically demanding because of the requirements to capture the large attenuation differences between lungs and mediastinum, to depict small contrast differences and to present fine structural details. Imaging these intrinsic properties of pulmonary tissues requires detectors with a wide dynamic range in order to capture the attenuation differences. High contrast detectors are essential to resolve lesions with subtle contrast differences emanating from their attenuation properties. These detectors should also have high spatial resolution in order to be able to display interstitial lung disease [9]. Therefore, achieving and maintaining high image quality in radiography is essential in order to ensure accurate diagnosis and effective patient management [10,11]. Poor image quality can obscure these details, leading to diagnostic errors or the need for additional imaging.

The assessment of the image quality (IQ) of digital systems is typically undertaken with physical quality metrics, such as modulation transfer function (MTF), noise power spectrum (NPS), detective quantum efficiency (DQE), contrast-to-noise ratio (CNR), and signal-difference-to-noise ratio (SDNR). Although these parameters objectively describe the inherent performance of the imaging detectors, it is difficult to link them to clinical image quality [12]. To help overcome this, a new IQ metric has been developed, named the “detectability index” (d′). This index relates objective measurements, such as the SDNR and the MTF, to actual clinical interpretation tasks. Through d′, IQ evaluations derived with a simple phantom can be directly linked to clinical imaging performance.

Effective quality control (QC) ensures acceptable image quality and reduces the likelihood of repeat examinations due to poor image quality, thereby minimizing unnecessary radiation exposures to patients. This is particularly important in lung imaging studies, where patients may require multiple imaging studies over time. The International Atomic Energy Agency (IAEA) has developed a novel approach for streamlining image quality (IQ) testing in digital radiography (DR) [13]. This method, designed to enhance the testing frequency, utilizes an affordable, easy-to-manufacture phantom alongside complimentary, no-cost software for the analysis of the captured images. The IAEA’s comprehensive package includes several resources, such as (1) detailed blueprints for the phantom, enabling precise construction; (2) the Automated Tool for Image Analysis (ATIA) software, which automatically assesses the phantom images and generates detailed image quality metrics which can be outputted in an Excel format; (3) an Excel file template for systematically recording IQ test results and generating long-term performance graphs for each radiographic facility; and (4) a comprehensive user manual for the ATIA software [13,14]. The IAEA method underwent a validation study conducted by the expert group that developed the approach. Initial findings indicated that the phantom is straightforward to produce and that the suggested method enables frequent QC tests—on a daily or weekly basis. This is made possible by the software’s ability to fully automate the evaluation of key performance aspects of the imaging chain [14].

In 2021, the IAEA launched a comprehensive five-year Coordinated Research Project (CRP) entitled “Advanced Tools for Quality and Dosimetry of Digital Imaging in Radiology” (CRP E24025). The primary objective of CRP E24025 was to evaluate the feasibility and effectiveness of implementing the IAEA methodology in radiology centers globally, encompassing a variety of radiological environments. This initiative aimed to enhance clinical practice by improving IQ and dosimetry standards. The project engaged 11 institutions from diverse countries worldwide, representing both well-resourced and less-resourced healthcare systems. The participating countries included Argentina, Brazil, France, Greece, Hungary, Ireland, Mexico, Malaysia, Qatar, Slovenia, and Sudan. Through this global collaboration, the IAEA sought to standardize and elevate the quality of radiological services, ensuring better diagnostic outcomes and patient care across different clinical settings. The participating institutions agreed on a comprehensive and collaborative work plan designed to rigorously test the IAEA methodology. This work plan included an initial pilot survey that was conducted over a six-month period, involving a smaller number of radiography systems. The pilot survey was designed to collect preliminary data and evaluate the practicalities, challenges, and benefits of the IAEA’s approach to quality and dosimetry in digital radiography, which is highly relevant for imaging of lung diseases. Overall data collection extends beyond the initial six months period and will proceed until the conclusion of the project at the end of 2026. It is also envisaged to recruit more participants.

Eight of the eleven participating institutions submitted digital radiography data sets acquired during the pilot survey phase. This paper presents the results of the initial survey, highlighting the variations observed in different radiological settings and the effectiveness of the implemented methodologies. The paper also investigates the possibility of setting minimum performance levels that could be universally applicable. The applicability and effectiveness of the methodology are crucial for assessing the performance of X-ray equipment used in lung disease investigations worldwide, thereby enhancing the diagnostic accuracy and the management of pulmonary conditions.

## 2. Materials and Methods

### 2.1. Radiological Equipment and Acquisition Protocol

The overall coordinated research project enrolled a total of 35 digital radiography machines from various manufacturers. These X-ray units are installed in diverse healthcare facilities in 11 participating countries (Argentina, Brazil, France, Greece, Hungary, Ireland, Mexico, Malaysia, Qatar, Slovenia, and Sudan).

However, for the pilot study, participants from only eight countries submitted image quality data, which had been derived from a total subset of 22 digital radiography units. The contributions are detailed in Section 3 (“Results”).

All of the systems included in this study were subjected to regular performance testing as part of the local quality assurance program requirements, and all of relevant parameters of these X-ray units were within the adopted performance limits. All units were direct digital radiography systems. The tube current-exposure time product was selected either manually or automatically by the automatic exposure control (AEC) system.

The image acquisition protocol is summarized in Table 1. For the pilot 6-month survey, participants either applied this recommended technical protocol or protocols available in their X-ray units that were as close as possible to the agreed one. In X-ray units equipped with AEC systems, the central AEC cell and clinical abdomen technical protocol were used in order to ensure harmonization of the methodology across different institutions and equipment.

If available, “for processing” (i.e., raw) images were analyzed. In cases in which raw images could not be obtained, “for presentation” (i.e., processed) images were used for the analysis. Harvesting of raw images requires “service mode” access to an X-ray unit. Since this access was not available to all participants, images had to be retrieved via a picture archiving and communication system instead, providing processed images, only.

### 2.2. Phantoms

**Figure 1:**
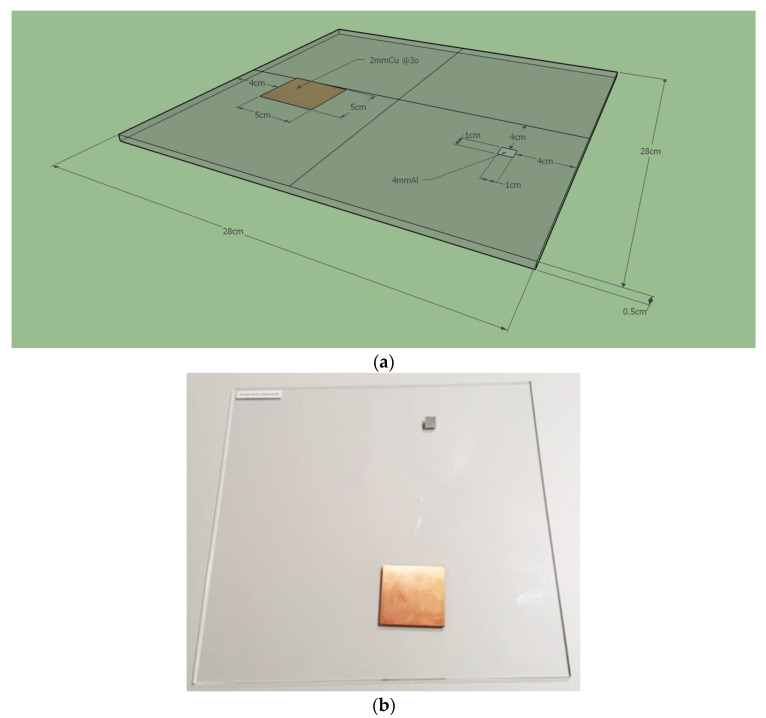
Presentation of (**a**) a schematic diagram of the radiography phantom and attenuators (reproduced from [13] with permission) and (**b**) a photograph of the phantom.

A brief outline of the phantom design, image acquisition parameters, and data pipeline are given below. The phantom used for remote and automated QC is considered simple and inexpensive [13,14]. The participating institutions manufactured their phantoms locally. As presented in Figure 1, the radiography phantom consists of a 5 mm thick polymethylmethacrylate (PMMA) plate that serves as a carrier and two rectangular inserts. The larger insert is a 5 cm × 5 cm and 0.2 cm thick copper (Cu) target plate intended for MTF and detectability index d′ analysis. The smaller insert is a 1 cm × 1 cm and 0.4 cm thick aluminum (Al) target plate enabling the derivation of signal-to-noise ratio (SNR), SDNR and d′ metrics. Detailed phantom specifications are provided in the IAEA Publication on the implementation of a remote and automated quality control program for radiography and mammography equipment [13].

### 2.3. Image Analysis and Image Quality Evaluation

Acquired phantom images were automatically analyzed by participating institutions using a dedicated software tool called ATIA. ATIA derives the IQ metrics’ values from the images and retrieves additional parameters from the associated image file Digital Imaging and Communications in Medicine (DICOM) headers, as listed in the left column of Table 2. Details on the computation of the IQ parameters are provided elsewhere [5]. In summary, the IQ metrics calculated by the ATIA software include spatial resolution, expressed as the MTF values at 50%, 20%, and 10% modulation (both horizontally and vertically), the SNR, the SDNR, and d′. Detectability index d′ metrics were calculated for the following two specific detection tasks: detection of conceptual circular details with diameters of 0.3 mm and 4 mm; contrast measured using the 0.4 cm thick Al target square. Additionally, ATIA reports five DICOM header values related to X-ray exposure including tube potential (kVp), tube loading (mAs), as well as the dose metrics, organ dose, entrance dose, and exposure index (Table 2). The ATIA IQ metrics values are exported to an Excel file template (‘Running Charts Generator’) for recording, archiving, and data visualization purposes. Each Excel file represents longitudinal IQ data for a single, specific radiographic unit.

## 3. Results

This section reports first the experiences made with the phantom construction activity before focusing on the results of the pilot study.

A total of 54 phantoms were constructed by 11 participating institutions for the overall coordinated research project. Table 3 details the number of phantoms produced by all participants and the respective costs. The experiences gained during the phantom construction process confirmed the simplicity of the process since the participants did not report any major challenges. The blueprints provided along with the IAEA method manual appeared to be clear and easy to follow. The phantoms consisted of low-cost material, readily available in all countries but Sudan. The use of locally obtainable materials facilitated reduction in costs and logistical challenges, making the phantoms a practical solution for implementation. In Mexico, the challenge was to identify the most cost-effective technique to produce copper inserts of the required size that would ensure acceptable repeatability in IQ metrics. Two construction procedures were discarded. First, guillotine cutting of the copper insert was abandoned as it increased the coefficient of variation (CV) of the MTF by 20%. Second, stacking two sheets of copper in the radiography phantom was rejected because of a similar increase in the CV of the MTF. The key lesson learned was that improving the quality of the copper edges reduces most of the inter- and intra-phantom variability. Some countries reported that the exact material (Al or Cu) thicknesses were difficult or impossible to find. In Hungary, it was not possible to obtain 0.2 mm thick Al, and the closest thickness was 0.3 mm. In Sudan, it was difficult to find 0.2 mm Al sheets, so Al cooking foil was used with two layers of 0.1 mm thickness, instead. Construction costs ranged from EUR 25 to 100 per phantom.

For the purpose of the pilot study, eight participating countries (Argentina, Brazil, France, Greece, Ireland, Malaysia, Mexico, and Qatar) collected and submitted image quality data. The data were gathered during weekly QC testing between April and December 2022, which was the agreed upon duration of the pilot study.

The contributions of each participant are detailed in Table 4. In total, 22 radiography units and 36 digital image detectors were employed in the study. Since the digital detectors could be used with different radiography units and in various setups (chest bucky, table bucky, and free) the total number of data sets acquired exceeded the respective numbers of radiography units and detectors. In the terminology used, “data sets” represent longitudinal data of measured image quality parameters. A total of 47 longitudinal data sets were derived in the study, denoted with unique identifiers IDs #1 to #47, as listed in the last column of Table 4. The term “Exposures” corresponds to single phantom exposures. The digital images were acquired for the subsequent generation of IQ parameters with the ATIA software. In total, 968 exposures were performed in order to establish a database of image quality metrics.

In support of the IQ data participants submitted metadata describing the radiographic setup, technique factors, and imaging modes used. The technique factors are extracted from the header information of the DICOM images. The numerical and categorical metadata collected for each unit are summarized in the two right columns of Table 2. Scripts were developed in the Python programming language to collate the IQ data and associated metadata into a single database and to allow for the conditional visualization of IQ data as functions of metadata.

DICOM header observations from the study highlighted several important issues. Firstly, the reporting of required DICOM tags was found to be vendor-dependent, with some vendors not including crucial tags, such as the exposure index and entrance dose. Additionally, DICOM values, such as mAs, were sometimes rounded or even set to zero, which could affect the precision of the data. It was also observed that the organ dose was consistently reported as zero. Furthermore, discrepancies were noted in the tags used for certain parameters, such as the exposure index, which differed from the tags the ATIA software searches for. Entrance dose and exposure index data were reliably given in only 43% of all the data, mainly because of nonuniversally valid DICOM tag addresses. Thus, entrance dose and exposure index data were excluded from the analysis due to their scarcity.

**Figure 2:**
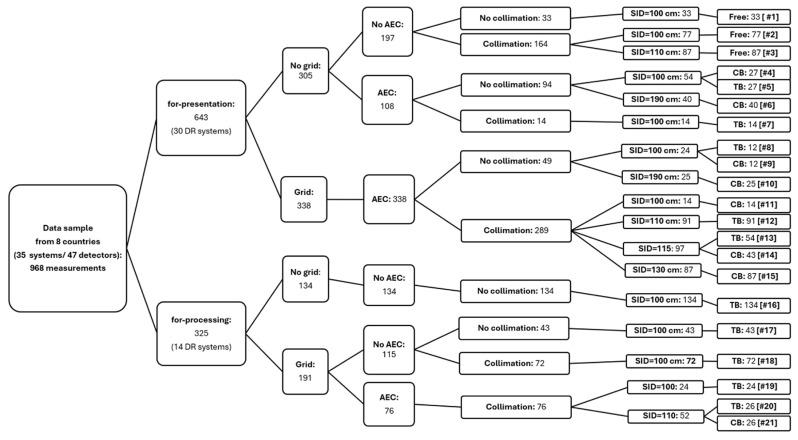
Distribution of the pilot study into different cohorts based on the image type (raw or processed) and the acquisition conditions (grid: yes/no; AEC: yes/now; collimation: yes/no; TB/CB/free and SID) used.

First, the variability observed in the metadata is described; that is, the range of exposure parameters utilized by the participants for phantom imaging is presented. This is followed by an analysis of the range of IQ parameters acquired and how its variability is affected by the choice of set-up factors. The scale of set-up factor variability used throughout the pilot study is illustrated as a tree plot in Figure 2. However, it must be noted that set-up factors were kept constant on each radiographic unit for the phantom imaging and that the variability exhibited in Figure 2 represents differences among individual radiographic unit/digital detector combinations used by the participants.

Figure 2 shows that 65% of the image quality data were derived from processed images (i.e., “for presentation”), while 35% of the IQ data were based on the analysis of raw images (i.e., “for processing”). Although the IAEA method recommends the use of raw images to avoid the influence of postprocessing algorithms on IQ metric values, the clinical reality often makes this challenging. Obtaining raw images from X-ray systems is difficult because many manufacturers either restrict access to these images or make them hard to obtain. Further detailed are the relative contributions of the remaining set up parameters, that is, scattering grid: yes/no; AEC: yes/no; collimation: yes/no; SID; and detector position.

For processed images, the grid usage was divided into 47% without a grid and 53% with a grid. Among those without a grid, 65% lacked an AEC, while 35% included an AEC. All instances with a grid had an AEC applied. The collimation varied between 27% without collimation and 73% with collimation for the non-AEC set-up cohort, and 24% without collimation and 75% with collimation for the AEC set-up cohort. The SID ranged from 100 cm to 190 cm; detector positions were detailed as chest bucky (CB), table bucky (TB), and free.

For raw images, the grid usage was split into 41% without a grid (mainly for mobile X-ray systems) and 59% with a grid. All instances without a grid lacked an AEC and collimation. Among the grid setups, 60% lacked an AEC, and 40% included an AEC. Collimation was used in 38% without an AEC and in all AEC setups. The SID and detector positions were dominated by SIDs of 100 cm and table bucky (TB) use, respectively.

The data indicated that, although the initial implementation of a common protocol seemed straightforward, clinical practice revealed particular challenges. It became apparent that even systems of the same model had varying clinical setups in different institutions, complicating the standardization process. This variability necessitated the development of more flexible and adaptable quality control methodologies to accommodate the diverse configurations observed in real-world settings.

The IQ data were clustered into 21 cohorts, each representing identical set-up conditions. The cohorts were denoted by unique identification numbers, Cohort IDs #1 to #21, as indicated in brackets in the last column of Figure 2. Their sample sizes ranged from N = 12 to N = 134 (last column of Figure 2).

With the terminology used throughout the paper, data set IDs #1 to #47 denote particular radiography units/imaging detector combinations or “DR systems” used by individual participants while the Cohort IDs #1 to #21 group data with identical imaging acquisition conditions.

The results of the image quality measurements are summarized in Table 5, giving the range (min, max), median, mean, and standard deviation for each parameter over the entire database but also separately for the processed images and raw images cohorts. Variability in the construction of the phantom, such as inconsistencies in Cu cutting or machine filing, can lead to large fluctuations in MTF values. Similarly, exposure variability can cause wide variations in the SDNR and SNR values. As shown in the table, the SNR values exhibit much greater variability compared to other IQ metrics, making SNR a less reliable indicator of image quality due to its susceptibility to variations in the setup and exposure conditions.

**Figure 3:**
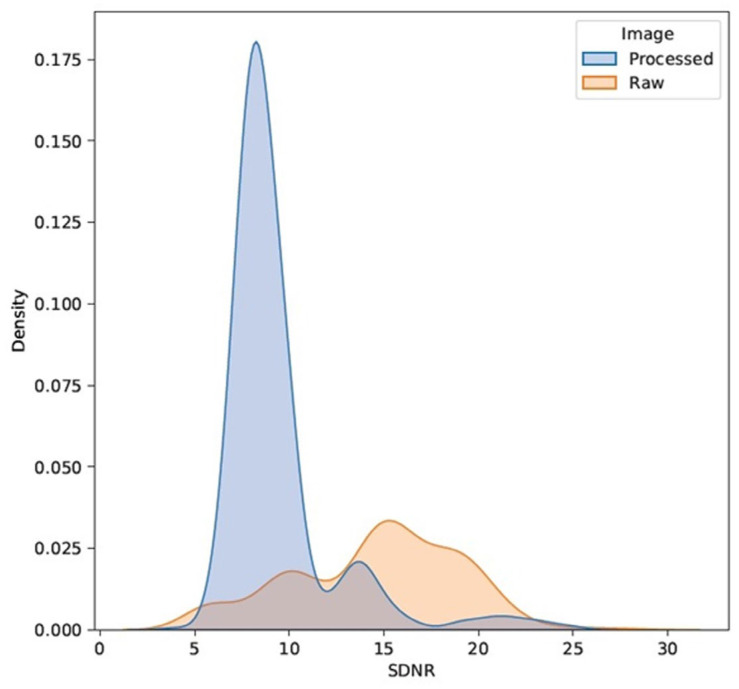
Density distributions of the SDNRs for the processed/raw image cohorts.

**Figure 4:**
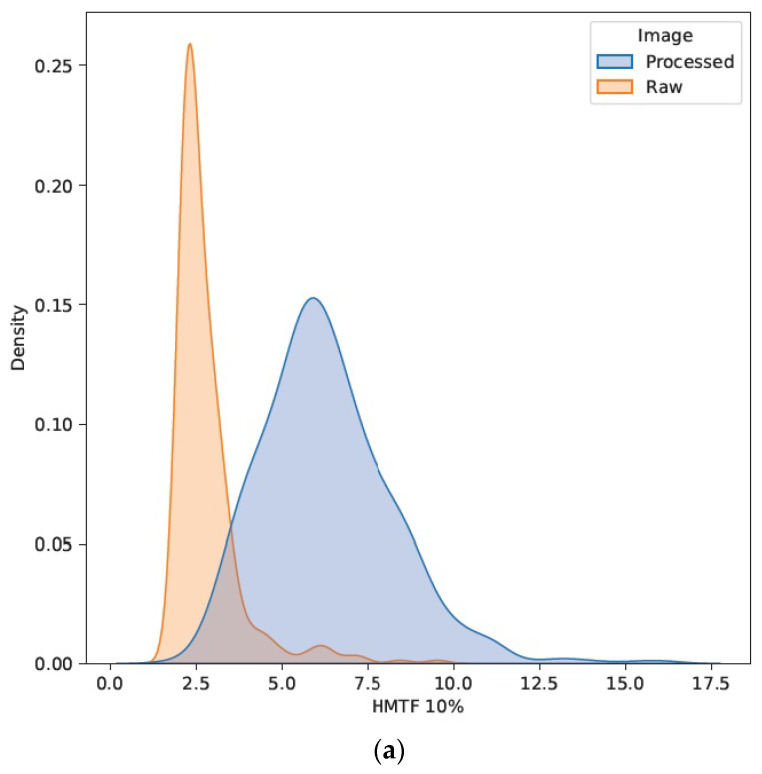

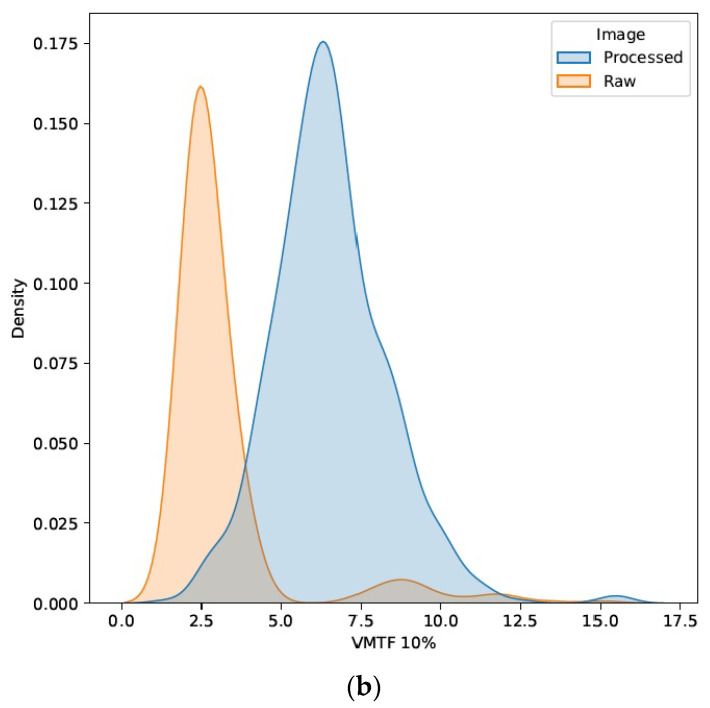
Density distributions of the (**a**) horizontal and (**b**) vertical 10% MTFs (in lp/mm) for the processed/raw image cohorts.

The large magnitudes and variations in the image quality parameters are further exemplified for SDNR, as well as the horizontal and vertical 10% MTFs, in Figure 3 and Figure 4, respectively. These figures show the density distributions smoothed with a Gaussian kernel for visualization purposes with a color-coded separation of the two major data cohorts of processed and raw images. Two distinct modes are apparent as a function of image processing, as was observed in the residual IQ parameters considered in this study (not graphed).

In order to eliminate the contribution of incorrect phantom designs to the variability observed in the acquired IQ parameters, the SDNR and MTF data were pre-analyzed. Specifically, the use of Al and Cu target thicknesses deviating from recommended specifications might affect the measured image quality.

**Figure 5:**
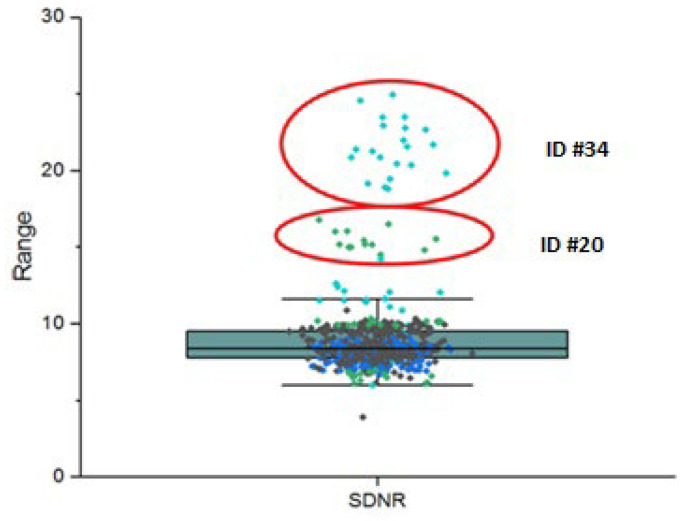
Box plot of the SDNRs for the processed images cohort. Outliers in the data and associated data set IDs are indicated.

The signal-difference-to-noise ratios are derived from the Al target plate of the phantom thus, the box plot in Figure 5 summarizes the SDNR data of the processed images cohort. The colors in the figure denote SDNR values of different data sets. The plot indicates two groups of outliers, that is, data points exceeding 1.5 IQR (interquartile range) above the third quartile. The two groups are encircled and are associated with the data set IDs #20 and #34 (cf. Table 4) of two participants. Closer examination revealed that due to supply issues Al targets of 4.8 mm instead of 4.0 mm thicknesses had to be used for phantom construction in this case causing an increase in SDNR values.

**Figure 6:**
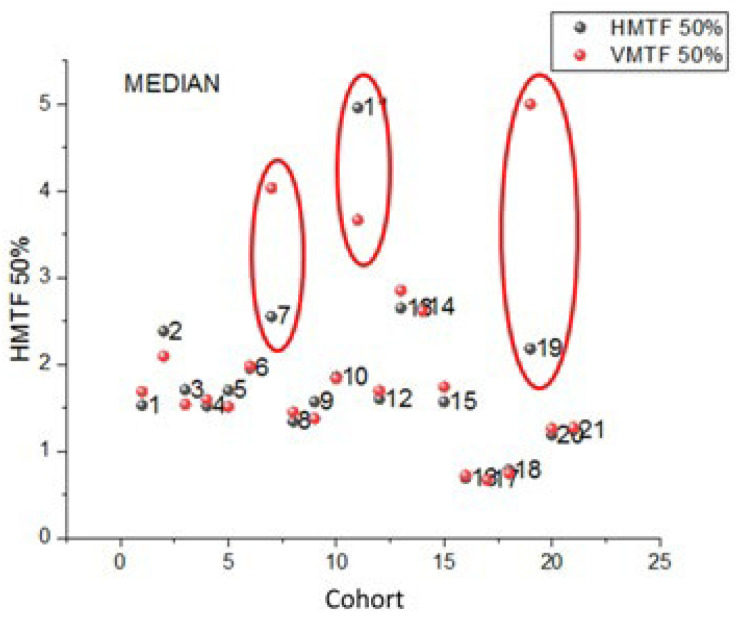
Deviations between the medians of the 50% horizontal and vertical MTFs for all 21 cohorts.

In order to evaluate the use of Cu targets, deviations between the medians of the 50% horizontal and vertical MTFs are plotted in Figure 6 for all 21 cohorts (cf. Figure 2). The horizontal and vertical MTFs are color-coded in black and red, respectively; Cohort IDs are added to the data points. In comparison, the encircled Cohort IDs #7, #11, and #19 exhibit significant differences between their horizontal and vertical MTF values (all *p*-values < 0.01, paired *t*-test). Cohorts #7, #11, and #19 are each represented by a single data set with IDs #7, #6, and #5, respectively.

After the completion of the pilot study blunt edges of the Cu inserts used for deriving the data sets of IDs #7 and #6 were identified as a cause for the large MTF differences between the horizontal and vertical MTF values.

Thus, the data sets of IDs #5, #6, #7, #20, and #34 were excluded from further analysis of the MTF values, which is thus based on 42 data sets in total.

**Figure 7:**
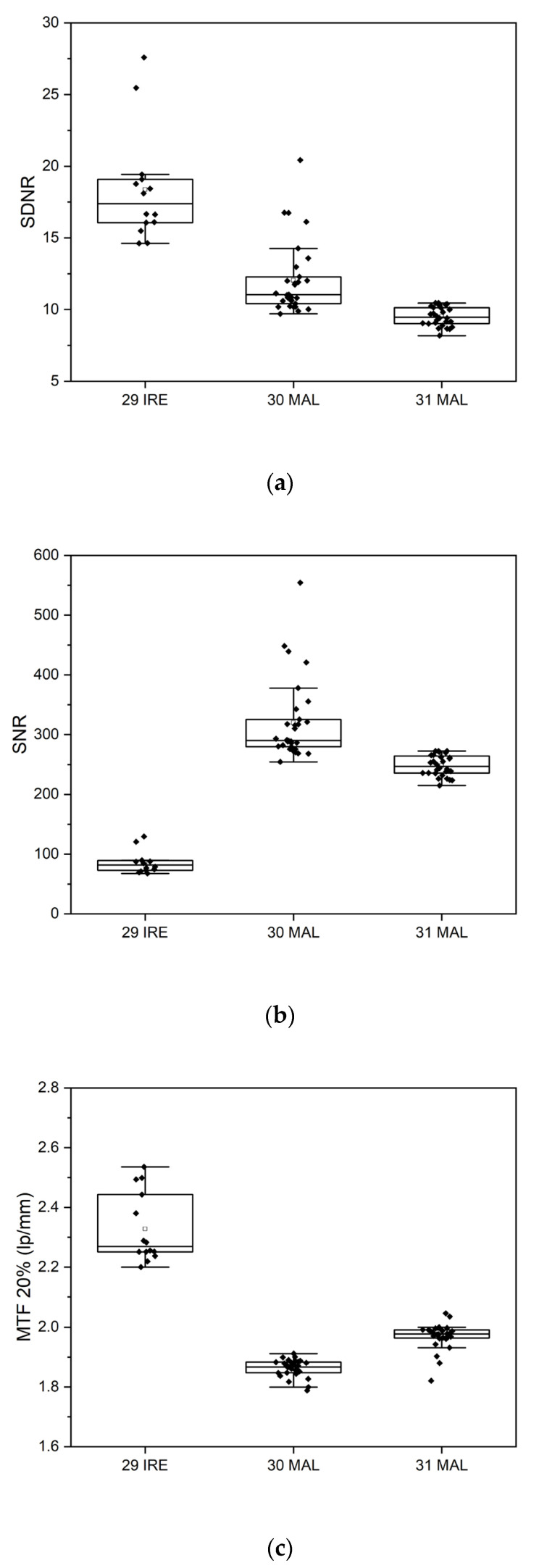
Box plots for the comparison of (**a**) SDNR, (**b**) SNR, and (**c**) MTF20% for data sets #29, #30, and #31.

In order to investigate the effect of radiographic set-up factors on the variability of IQ metric values, data sets had to be identified that were obtained on radiographic units of the same vendor and also with the same image acquisition parameters. Three data sets acquired under similar conditions could be identified. Box plots exemplifying a comparison of the SDNR, SNR, and MTF 20% values for these data sets are displayed in Figure 7. Evident is the discrepancy in IQ parameters between data set #29 and data sets #30 and #31, despite comparable imaging conditions. Data set IDs #30 and #31 were submitted by the same participant.

**Figure 8:**
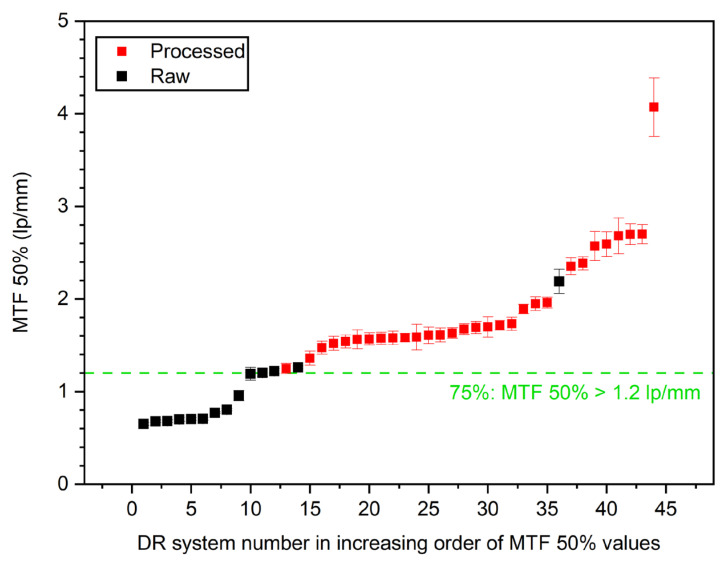
Distribution of horizontal MTF 50% values.

**Figure 9:**
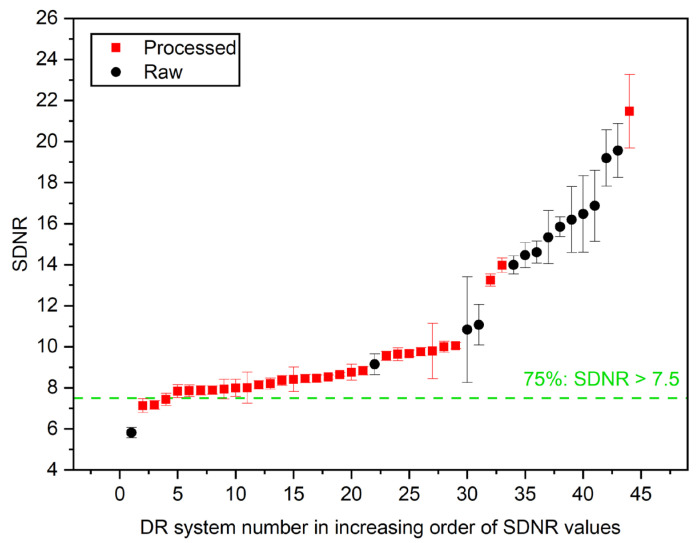
Distribution of the SDNR values.

**Figure 10:**
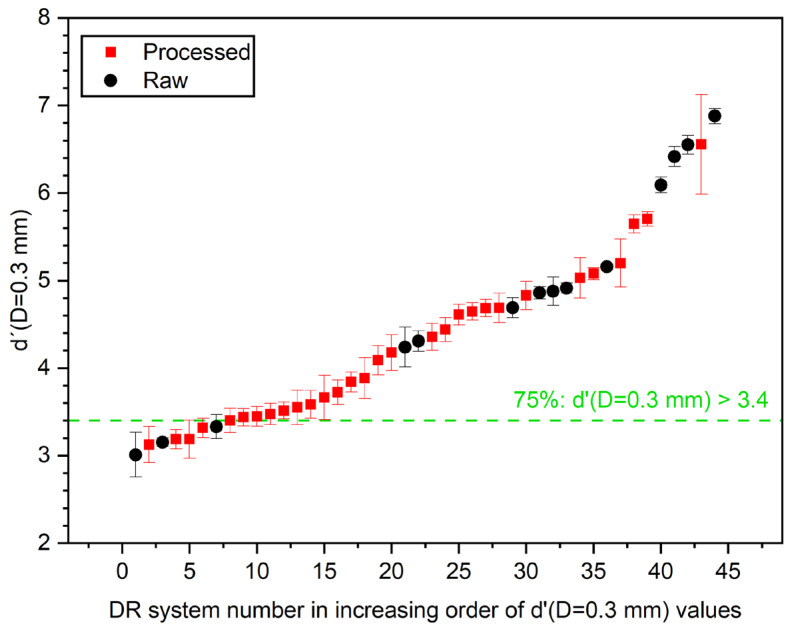
Distribution of d′ (D = 0.3 mm) values.

Finally, to investigate the possibility of setting minimum performance levels that could be universally applicable, the distribution of horizontal MTF 50%, SDNR, and d′ were plotted in Figure 8, Figure 9 and Figure 10, respectively, for each data set, representing a unique radiography unit/detector combination or “system” (cf. Table 4).

In Figure 8, horizontal MTF 50% values are sorted in increasing order. In the figure, the raw-image-derived (black dots) and processed-image-derived (red squares) MTF 50% values are compared across different DR systems. The horizontal green dashed line represents the threshold at which 75% of the systems have an MTF 50% value greater than 1.2 line pairs per millimeter (lp/mm). Key observations from the graph include that processed images generally exhibit higher MTF 50% values compared to raw images and, thus, confirming that postprocessing enhances the perceived spatial resolution. Furthermore, there is a notable variability in MTF 50% values across the data sets, with some DR systems showing much higher values than others, reflecting differences in the system performance and image processing algorithms. The error bars on each data point indicate the range of variability or uncertainty in the measurements, further highlighting the differences between raw and processed image quality metrics.

Figure 9 displays the SDNR values sorted in increasing order, comparing raw-image-derived (black dots) and processed-image-derived (red squares) SDNR values across different DR systems. The horizontal green dashed line represents the threshold at which 75% of the systems have an SDNR value greater than 7.5. As shown in the graph, the processed images generally exhibited lower SDNR values compared to the raw images across most DR systems, indicating that postprocessing may reduce the apparent signal difference relative to noise.

Figure 10 displays the detectability index, d′, for a virtual disk with a diameter of 0.3 mm, sorted in increasing order. Again, the graph compares the d′ derived from raw images (black dots) and processed images (red squares) across different DR systems. The horizontal green dashed line represents the threshold at which 75% of the systems have a d′ value greater than 3.4. The raw images generally exhibited higher d′ values compared to the processed images across most DR systems, indicating that raw images may better reflect the detectability of small details.

As shown in the figures above, postprocessing appears to enhance spatial resolution (horizontal MTF 50%), with 75% of the systems achieving values over 1.2 lp/mm. However, while detectability (d′) also improved, with 75% of the systems exceeding a value of 3.4, the SDNR values, which measure contrast, are slightly lower for processed images compared to raw images. Seventy-five percent of systems exceeded an SDNR threshold value of 7.5.

## 4. Discussion

Remote QC enhanced with automated software can play a critical role in maintaining consistent and high-quality radiographic imaging [15,16]. This is particularly important in chest X-rays, where precise imaging is not only crucial for diagnosing conditions, such as lung cancer, pneumonia, and tuberculosis, but also in the management of screening centers where stringent tolerances and high image quality are paramount. Annual testing alone is insufficient for detecting short-term fluctuations in critical components of the imaging chain; thus, more frequent testing is necessary. Remote QC testing provides a practical solution to maintain consistency and reliability among comprehensive evaluations. This approach helps ensure that any minor variations are promptly identified and addressed, supporting the overall integrity and performance of the imaging systems [5]. If remote QC testing employs automated software, more advanced analyses can be performed by evaluating entire images, which otherwise would not be feasible with manual tests focusing on localized measurements. This approach can enhance QC testing frequency, accuracy, and efficiency, ensuring that image quality remains consistent among comprehensive annual evaluations [17,18,19]. The IAEA remote and automated methodology [5] was initially validated by two members of the group of experts that developed the method through tests in their specific clinical scenarios [14]. It was also tested extensively by one participating institution from Qatar that concluded that the IAEA phantom demonstrates advantages over sophisticated commercial phantoms which are also accompanied by dedicated software [20]. According to the study, the main IQ metrics of the IAEA phantom (like SDNR and d′) followed the expected trends with kV and dose variations while commercial phantoms failed to do so, most probably because processing has masked these changes in their derived IQ metrics [20].

In the current research study, the method was applied in wide clinical scenarios by institutions that were not involved in its initial development and that exhibited diverse educational backgrounds and health systems. It is the first attempt of a wide-scale implementation of the IAEA method in diverse clinical settings across the world. As such, it is not possible to make direct comparisons of this extensive implementation with existing literature since no prior studies have addressed this level of global application. This unique approach aims to provide comprehensive insights and aims to establish a new benchmark for radiographic quality control in varied healthcare environments.

The pilot survey results revealed a number of important issues that need to be addressed for further implementation in the next phase of the study, which will continue until the end of 2026, as follows:

(1) Although the IAEA method recommends using raw images to avoid the influence of postprocessing algorithms on IQ metric values, implementing this in clinical practice is often challenging. Many manufacturers restrict access to raw images or make them difficult to obtain, hindering consistent application of the recommended protocols.
(2) Additionally, it became apparent that even X-ray systems of the same model can have varying clinical setups, further complicating the standardization process. This variability in configurations across different clinical environments underscores the need for adaptable quality control methodologies to ensure consistent image quality. Even X-ray systems of the same vendor used with identical clinical setups and protocols can exhibit variations in the calculated IQ metric values, as shown in Figure 7.
(3) Variations in the construction of the phantom, such as inconsistencies in Cu target plate cutting or machine filing, can lead to large fluctuations in MTF values (CV > 20%), see Figure 6. These construction inconsistencies introduce a range of errors, affecting the accuracy and reliability of MTF measurements, which are crucial for assessing spatial resolution in radiographic imaging.
(4) The reporting of required DICOM tags in the image file headers was found to be vendor-dependent, with some vendors not including crucial tags or occasionally misrepresenting tag values, such as mAs, which were sometimes rounded or even set to zero. Discrepancies were noted in the tags used for certain parameters, such as the exposure index, which differed from the tags that the ATIA software searches for.
(5) ATIA is an executable GUI-based software that requires human interaction to produce test image results. This necessity for manual operation poses a challenge to achieving full automation in this step. In the next phase of the coordinated research project, a complete automation of the image handling process could be investigated. This will minimize the workload for all participating individuals, including medical physicists and radiographers.
(6) The SNR values exhibit much greater variability compared to the other IQ metrics. This high degree of fluctuation makes the SNR a less reliable indicator of overall image quality. The large variations in the SNRs can obscure meaningful comparisons and trends, leading to potential misinterpretations of imaging performance. In contrast, metrics like SDNR or d′ tend to show better consistency, providing a more dependable basis for evaluating the quality of radiographic images.
(7) Figure 8, Figure 9 and Figure 10 indicate that the minimum performance thresholds for DR systems could be set at 1.2 lp/mm for horizontal MTF 50%, at 7.5 for SDNR, and at 3.4 for d′. These preliminary benchmarks need further investigation in the next project phase in order to determine their universal applicability. For example, for AEC-operated systems, a lower SDNR value may be an indication of setting a very low switch-off dose at the AEC system sensors (i.e., well below 2.5 μGy).

In conclusion, cross-comparisons among different radiography systems must be approached cautiously because of the sensitivity of the methodology to variations in phantom construction and acquisition mode. This important lesson from the survey highlights that differences in how the phantom is built and differences in the specific settings used for image acquisition can influence the results. While the IAEA framework is beneficial for evaluating image quality (IQ) properties, especially for specific applications like chest imaging, users must be aware of these sensitivities. This awareness is crucial to ensure accurate and fair comparative evaluations among different units or facilities, thereby promoting more reliable and standardized QC practices in radiography. This is particularly important in chest imaging due to the high frequency of this type of examination.

## Figures and Tables

**Table 1:** Radiography image acquisition protocol.

Attenuator plate: 2 mm Cu filter (10 cm × 10 cm)
Focus-to-detector distance: 100 cm
Beam collimation to the phantom size corresponding to the size of the PMMA plate
Tube potential: 80 kVp (practical peak voltage)
Tube current–exposure time: 20 mAs for systems without AEC
Anteroposterior abdomen protocol for systems with AEC (AEC central cell only)

**Table 2:** Database of image quality metrics estimated by the Automated Tool for Image Analysis (ATIA) software, and metadata.

ATIA Derived IQ Metrics	Metadata	DICOM Header Information
Signal-to-noise ratio (SNR)	Vendor/model name	Tube current–exposure time: mAs
Signal-difference-to-noise ratio (SDNR)	Image receptor placement: Chest bucky/table bucky/free	Tube peak potential applied: kVp
Horizontal modulation transfer function (MTF) @ 50%, 20%, and 10%	Image receptor dimensions (cm × cm)	Entrance dose (ED)
Vertical modulation transfer function (MTF) @ 50%, 20%, and 10%	Image type: raw/processed	Exposure index (EI)
Detectability index (d′) for a disk with diameter, D = 0.3 mm	Scattering grid used: yes/no	
Detectability index (d′) for a disk with diameter, D = 4.0 mm	AEC applied: yes/no	
	Imaging protocol: pattern/abdomen/chest	
	Beam collimated to target plate: yes/no	
	Focus-to-detector distance (cm)	

**Table 3:** Radiography phantom construction: total number of and cost per phantom in each country.

N	Country	Cost/Per Phantom (EUR)	N Phantoms
1	Argentina	49	3
2	Brazil	45	1
3	France	60	1
4	Greece	65	1
5	Hungary	50	4
6	Ireland	50	1
7	Malaysia	30	2
8	Mexico	25	3
9	Qatar	20	25
10	Slovenia	50	10
11	Sudan	100	3
	Sum		54

**Table 4:** Data contributions of the participants to the radiography pilot study.

Country	N Radiography Units	N Digital Detectors	N Data Sets	N Exposures	Data Set IDs
Argentina	3	3	3	46	#33, #34, #37
Brazil	4	5	14	320	#1–4, #25–28, #38–43
France	1	2	4	52	#12–15
Greece	1	1	1	24	#5
Ireland	1	1	1	14	#29
Malaysia	1	2	2	58	#30, #31
Mexico	2	3	3	41	#6, #7, #20
Qatar	9	19	19	413	#8–11, #16–19, #21–24, #32, #35, #36, #44–47
Sum	22	36	47	968	

**Table 5:** Statistical summary of the results of the image quality measurements for the entire data set, as well as the cohorts of processed and raw images.

	Min	Max	Median	Mean	StDev
All Images					
SDNR	3.909	27.591	9.427	11.127	4.258
SNR	4.927	649.783	27.584	123.124	178.068
HMTF 50%	0.626	5.744	1.592	1.640	0.836
HTMF 20%	1.482	8.468	3.160	3.278	1.322
HTMF 10%	1.763	16.166	5.143	5.133	2.357
VMTF 50%	0.596	5.583	1.613	1.736	0.997
VMTF 20%	1.243	10.147	3.439	3.480	1.490
VMTF 10%	1.360	15.720	5.623	5.409	2.458
d′ (D = 0.3 mm)	2.356	9.901	4.431	4.539	1.190
d′ (D = 4 mm)	39.819	189.093	80.082	84.679	29.379
Processed Images					
SDNR	3.909	24.935	8.484	9.382	2.944
SNR	4.927	319.026	14.063	24.214	34.833
HMTF 50%	1.004	5.744	1.701	2.008	0.752
HTMF 20%	1.613	8.468	3.797	3.939	1.070
HTMF 10%	1.763	16.166	6.090	6.315	1.925
VMTF 50%	0.919	5.550	1.750	2.041	0.763
VMTF 20%	1.243	9.124	3.877	4.074	1.014
VMTF 10%	1.360	15.720	6.404	6.549	1.792
d′ (D = 0.3 mm)	2.356	8.156	4.019	4.223	0.924
d′ (D = 4 mm)	39.819	122.010	70.281	73.046	16.556
Raw Images					
SDNR	5.325	27.591	15.034	14.579	4.352
SNR	43.250	649.783	325.257	318.813	185.481
HMTF 50%	0.626	2.453	0.715	0.913	0.412
HTMF 20%	1.482	4.553	1.643	1.969	0.599
HTMF 10%	2.044	9.535	2.327	2.795	0.988
VMTF 50%	0.596	5.583	0.733	1.133	1.124
VMTF 20%	1.496	10.147	1.726	2.306	1.584
VMTF 10%	1.971	15.087	2.432	3.155	2.001
d′ (D = 0.3 mm)	2.660	9.901	4.933	5.165	1.394
d′ (D = 4 mm)	42.117	189.093	101.515	107.696	35.113

## Data Availability

The original contributions presented in the study are included in the article, further inquiries can be directed to the corresponding authors.

## References

[1] Radiology ACo (2017). ACR-SPR-STR Practice Parameter for the Performance of Chest Radiography. https://www.acr.org/-/media/ACR/Files/Practice-Parameters/ChestRad.pdf.

[2] Veldkamp W.J., Kroft L.J., Geleijns J. (2009). Dose and perceived image quality in chest radiography. Eur. J. Radiol..

[3] United Nations Publications (2022). Sources, Effects and Risks of Ionizing Radiation, United Nations Scientific Committee on the Effects of Atomic Radiation (UNSCEAR) 2020/2021 Report, Volume I, Report to the General Assembly, with Scientific Annex A—Evaluation of Medical Exposure to Ionizing Radiatio.

[4] Candemir S., Antani S. (2019). A review on lung boundary detection in chest X-rays. Int. J. Comput. Assist. Radiol. Surg..

[5] Wielputz M.O., Heussel C.P., Herth F.J., Kauczor H.U. (2014). Radiological diagnosis in lung disease: Factoring treatment options into the choice of diagnostic modality. Dtsch. Arztebl. Int..

[6] Mathers C.D., Loncar D. (2006). Projections of global mortality and burden of disease from 2002 to 2030. PLoS Med..

[7] Fatihoglu E., Aydin S., Gokharman F.D., Ece B., Kosar P.N. (2016). X-ray Use in Chest Imaging in Emergency Department on the Basis of Cost and Effectiveness. Acad. Radiol..

[8] Kim T.J., Lee K.H., Choe Y.H., Lee K.S. (2021). Emergency Chest Radiology.

[9] Schaefer-Prokop C., Neitzel U., Venema H.W., Uffmann M., Prokop M. (2008). Digital chest radiography: An update on modern technology, dose containment and control of image quality. Eur. Radiol..

[10] International Atomic Energy Agency (IAEA) (2023). Handbook of Basic Quality Control Tests for Diagnostic Radiology.

[11] Tsapaki V. (2020). Radiation dose optimization in diagnostic and interventional radiology: Current issues and future perspectives. Phys. Med..

[12] Moore C.S., Wood T.J., Beavis A.W., Saunderson J.R. (2013). Correlation of the clinical and physical image quality in chest radiography for average adults with a computed radiography imaging system. Br. J. Radiol..

[13] International Atomic Energy Agency (IAEA) (2021). Implementation of a Remote and Automated Quality Control Programme for Radiography and Mammography Equipment.

[14] Mora P., Pfeiffer D., Zhang G., Bosmans H., Delis H., Razi Z., Arreola M., Tsapaki V. (2021). The IAEA remote and automated quality control methodology for radiography and mammography. J. Appl. Clin. Med. Phys..

[15] Jacobs J., Lemmens K., Nens J., Michielsen K., Marchal G., Bosmans H. (2008). One Year of Experience with Remote Quality Assurance of Digital Mammography Systems in the Flemish Breast Cancer Screening Program.

[16] Looney P., Halling-Brown M.D., Oduko J.M., Young K.C. (2015). A Pilot Study on the Development of Remote Quality Control of Digital Mammography Systems in the NHS Breast Screening Programme. J. Digit Imaging.

[17] Binst J., Verhoeven H., Lemmens K., Jacobs A., Jacobs J., De Wilde S., Marshall N., Bosmans H. 15 years of remotely controlled daily quality control in digital mammography. Proceedings of the 15th International Workshop on Breast Imaging (IWBI2020).

[18] Nowik P., Bujila R., Poludniowski G., Fransson A. (2015). Quality control of CT systems by automated monitoring of key performance indicators: A two-year study. J. Appl. Clin. Med. Phys..

[19] Visanuyanont T., Gluchowski P., Hillberg E., Moberg T., Svalkvist A. (2021). Automated Qc for Interventional Systems and Mammography Systems. Radiat. Prot. Dosim..

[20] Tsalafoutas I.A., AlKhazzam S., Tsapaki V., AlNaemi H., Kharita M.H. (2022). Digital radiography image quality evaluation using various phantoms and software. J. Appl. Clin. Med. Phys..

